# Involvement of TIP60 acetyltransferase in intracellular *Salmonella *replication

**DOI:** 10.1186/1471-2180-10-228

**Published:** 2010-08-26

**Authors:** Xueqin Wang, Dongju Li, Di Qu, Daoguo Zhou

**Affiliations:** 1 Department of Molecular Virology, Shanghai Medical College of Fudan University, Shanghai 200032, P.R. China; 2 Department of Biological Sciences, Purdue University, West Lafayette, IN 47907, USA

## Abstract

**Background:**

*Salmonella *enterica is a facultative intracellular pathogen that replicates within a membrane-bound compartment termed *Salmonella *containing vacuole (SCV). The biogenesis of SCV requires *Salmonella *type III protein secretion/translocation system and their effector proteins which are translocated into host cells to exploit the vesicle trafficking pathways. SseF is one of these effectors required for SCV formation and Intracellular *Salmonella *replication through unknown mechanisms.

**Results:**

In an attempt to identify host proteins that interact with SseF, we conduct a yeast two-hybrid screening of human cell cDNA library using SseF as the bait. We identified that TIP60, an acetyltransferase, interacts with SseF. We showed that the TIP60 acetylation activity was increased in the presence of SseF, and TIP60 was upregulated upon *Salmonella *infection. In addition, TIP60 is required for efficient intracellular *Salmonella *replication in macrophages.

**Conclusion:**

Taken together, our data suggest that *Salmonella *may use SseF to exploit the host TIP60 acetyltransferase activity to promote efficient *Salmonella *replication inside host cells.

## Background

Protein acetylation adds the acetyl group on either the amino-terminal residues or on the epsilon-amino group of lysine residues. Lysine acetylation affects many protein functions, including DNA binding, protein-protein interactions, and protein stability. TIP60 catalyzes histone acetylation [1,2]. It was originally identified as a cellular acetyltransferase protein that interacts with HIV-1 Tat [3]. Over-expression of TIP60 increased Tat transactivation of the HIV-1 promoter [3]. Recent studies found that TIP60 has diverse functions involved in transcription, cellular signaling, DNA damage repair, cell cycle checkpoint control and apoptosis [2,4,5].

*Salmonella enterica *serovar Typhimurium (*S. typhimurium*) causes gastrointestinal diseases in humans and typhoid-like fever in the mouse. *S. typhimurium *encodes two Type III secretion systems within the *Salmonella *pathogenicity islands 1 and 2 (SPI-1 and SPI-2) that are required for *Salmonella *entry and subsequent survival inside the host cells, respectively [6-10]. Following entry into the host cells, *S. typhimurium *replicates within a membrane-bound compartment termed *Salmonella*-containing vacuole (SCV). Previous studies have shown that SifA, SseF and SseG are involved in the formation of *Salmonella *induced filaments (Sifs) that are required for maintaining the SCV [11-13].

SseF, working together with SseG, has been shown to be involved in the aggregation of host endosomes and may help to position the *Salmonella*-containing vacuoles in close association with the Golgi network [14-19]. In the absence of SseF, the vacuolar compartments containing *Salmonella *were discontinuous and intracellular *Salmonella *replication was reduced [10,14,15,20-22]. SseG was shown to be co-localized with the trans-Golgi network and only bacteria closely associated with the Golgi network were able to multiply [11]. It has been shown that SseF interacts functionally and physically with SseG but not SifA and is also required for the perinuclear localization of *Salmonella *vacuoles [23]. The molecular mechanism on how SseF and SseG function remains unknown. In the present study, we set out to search the host target that interacts with SseF. We presented evidence indicating that *Salmonella *SseF interacts with TIP60 to potentiate its histone acetylation activity to promote intracellular replication.

## Methods

### Bacterial strains

Bacterial strains and plasmids used in this study are listed in Table 1. Chromosomal gene replacements were carried out by using a suicide plasmid [24,25]. *E. coli *and *S. typhimurium *strains are routinely cultured in Luria-Bertani broth (LB). *Salmonella *trains were grown in MgM minimal medium when SPI-2 TTSS-inducing conditions were desired [26]. Antibiotics used were: ampicillin at 120 μg/ml, streptomycin at 25 μg/ml, and tetracycline at 12 μg/ml.

**Table 1:** Bacterial strains and plasmids

Strains and plasmids	Relevant Characteristics	Source
***S. typhimurium and E. coli***		
SL1344	Wild-type *S. typhimurium*, Str^r^	[33]
ZF3	SseF in-frame deletions	This study
SM10 λ*pir*	*thi thr leu tonA lacY supE recA*::RP4-2-Tc::Mu (Kan^r^) λ*pir*	[34]
***Plasmids***		
pZP226	SsaV in-frame deletions in pSB890; Tc^r^	[20]
pZP227	SseF in-frame deletions in pSB890; Tc^r^	[20]
pZP784	SseF_Δ67-106, 161-174, 186-205 _in pGBT9, Ap^r^	This study
pZP2037	His-SseF in pET28a; Kan^r^	This study
pZP2038	His-SseG in pET28a; Kan^r^	This study
pZF1	GAL4AD-iTIP60_164-546 _in pGAD-GH; Ap^r^	This study
pZF2	GAL4AD-TIP60α in pGAD-GH; Ap^r^	This study
pZF3	GAL4AD-TIP60β in pGAD-GH; Ap^r^	This study
pZF4	HA-TIP60α in pcDNA3; Ap^r^	This study
pZF6	MBP-TIP60α in pIADL16; Ap^r^	This study
pZF8	GAL4-BD-SseF_1-66 _in pGBT9; Ap^r^	This study
pZF9	GAL4-BD-SseF_50-66 _in pGBT9; Ap^r^	This study
pZF10	GST-SseF_1-66 _in pGEX-KG; Ap^r^	This study
pZF11	GST-SseF_50-66 _in pGEX-KG; Ap^r^	This study
pZF280	GAL4-BD-SseF_1-56 _in pGBT9; Ap^r^	This study
pZF281	GAL4-BD-SseF_50-260 _in pGBT9; Ap^r^	This study
pZF282	GAL4-BD-SseF_1-228 _in pGBT9; Ap^r^	This study

### Mammalian cell lines and bacterial infection assay

The murine macrophage RAW264.7 (TIB-71, ATCC) and the human epithelial cell line HeLa (CCL-2, ATCC) were from the ATCC (Manassas, VA) and were maintained in Dulbecco's modified Eagle medium (DMEM) containing 10% FBS. Bacterial infection of RAW264.7 and survival assays were carried out using opsonized bacteria in DMEM containing 10% normal mouse serum as described before [10,20,27]. The extent of replication was then determined by dividing the number of intracellular bacteria at twenty-four hours by the number at two hours.

### Yeast Two-hybrid Screening

The GAL4-based yeast two-hybrid system was used following standard procedures [28]. The bait plasmid (pZP784) was constructed by deleting the putative three trans-membrane regions (67-106, 161-174, 186-205 a.a.) of SseF and fusing it to the yeast GAL4 binding domain in pGBT9.m [28]. A human cell cDNA library was constructed by oligo(dT) priming in pACT2 (Clontech Laboratories, Palo Alto, CA). A total of 5 × 10^5 ^transformants were screened in the yeast indicator strain AH109, using the sequential transformation protocol as described (Clontech Laboratories). Clones that grow on the yeast synthetic drop-out media lacking histidine and exhibited positive galactosidase on the X-Gal plates were chosen for further analysis.

### Protein purification and biochemical pull down assay

GST, His and MBP-tagged recombinant proteins were expressed and purified in *Escherichia coli *BL21 (DE3) using the pGEX-KG, pET28a, or the pMAL-c2x expression systems, respectively. The purification of the GST-tagged proteins was performed according to the manufacturer's instructions (Amersham, Pittsburgh, PA). Purified proteins were concentrated and buffer exchanged in PBS, using a 10 K and 30 K molecular weight cut-off dialysis cassette (Sartorius, Elk Grove, IL). Purified proteins were snap-frozen in liquid nitrogen and stored at -80°C in PBS/20% glycerol. Proteins were pre-clarified at 120,000 Xg, and their concentration was determined by Bradford assay (Bio-Rad) using bovine serum albumin as standard. Pulled-down proteins were analyzed by SDS-PAGE and Western blotting using appropriate antibodies. Western blots were developed with using the SuperSignal West Pico detection reagent according to the manufacturer's instructions (Thermo Fisher, Rockford, IL).

### HAT Assay

HAT assays were performed using recombinant MBP-TIP60 protein (100 ng) as acetyltransferase and histone (2 μg, Sigma, St. Louis, MO) as the substrate in 20 μl HAT buffer (50 mM Tris, pH 8.0, 10% glycerol, 1 mM dithiothreitol, 0.1 mM EDTA, 10 mM sodium butyrate) containing Acetyl-CoA (100 μM, Sigma, St. Louis, MO) for 30 min at 30°C. Acetylated histones were detected by Western blot, using the pan-acetyl antibody (Santa Cruz Biotech, Santa Cruz, CA).

### TIP60 siRNA

TIP60 siRNA expression plasmids were constructed in pSilencer 2.1 (Ambion, Austin, TX) with a pair of 63-bp oligonucleotides, each containing a unique 19-bp TIP60 sequence. For use in human cell lines: 5'-GATCCGAACAAGAGTTAATTCCCAGTTC AAGAGACTGGGAATAACTCTTGTTCTTTTTTGGAAA-3' and 5'-AGCTTTTCCAAAAAA GAACAAGAGTTATTCCCAGTCTCTTGAACTGGGAATAACTCTTGTTCG-3'. For use in mouse cell lines: 5'-GATCCAGACTGGAGCAAGAGAGGATTCAAGAGATCCTCTCTTGC TCCAGTCTTTTTTTGGAAA-3' and 5'-AGCTTTTCCAAAAAAAGACTGGAGCAAGAG AGGATCTCTTGAATCCTCTCTTGCTCCAGTCTG-3'. For negative control, a scrambled siRNA hairpin was placed into the same sites in pSilencer 2.1. These plasmids were transfected into cells using the siPORT *XP-1 *provided by Ambion. Transfected cells were maintained for 24 hours without selection; cultures were then subjected to G418 selection before infection.

## Results

### *Salmonella *SPI2 effector protein SseF interacts with TIP60 histone acetylase

**Figure 1:**
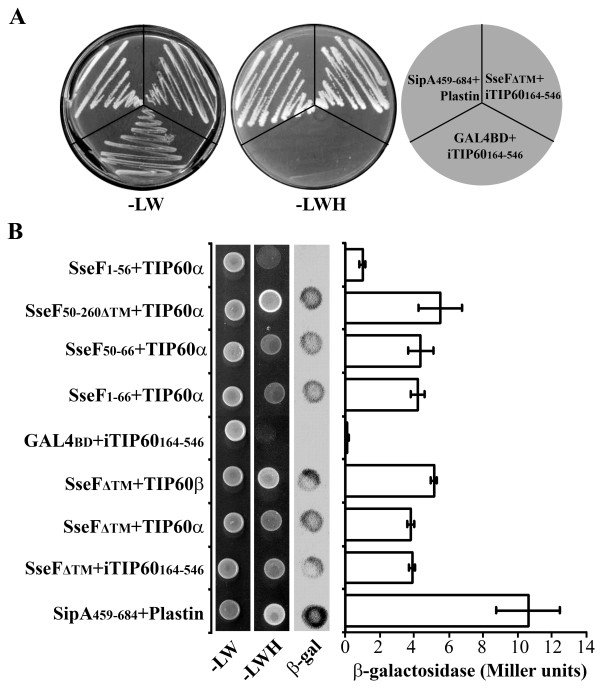
**Interaction of SseF with TIP60**. (A) Plasmids expressing the SseF devoid of putative transmembrane regions fused to the GAL4 binding domain were transformed into yeast strain AH109 expressing a fusion between the GAL4 activation domain and iTIP60^164-546 ^(pZF1). (**B**) Plasmids expressing the various SseF fragments fused to the GAL4 binding domain were transformed into yeast strain AH109 expressing a fusion between the GAL4 activation domain and different TIP60 isoforms. SipA together with Plastin was used as a positive control. Yeast strains expressing the above plasmid combinations were streaked on SD-Leu-Trp (-LW) or -Leu-Trp-His+15 mM 3-AT media (-LWH). Quantitative β-galactosidase activities were measured from yeast grown in SD-Leu-Trp and expressed in Miller units.

In a search for host proteins that interact with SseF, we conducted a yeast two-hybrid screening [29] of a human cell cDNA library, using a fusion of the DNA-binding domain of GAL4 and the truncated SseF devoid of transmembrane regions (pZP784, SseF_Δ67-106, 161-174, 186-205_) as the bait. One clone was identified which encodes the C-terminal 164-546 TIP60 histone acetyltransferase isoform 1 (Fig. 1). There are at least three splice variants of TIP60: TIP60 isoform 1 (iTIP60), TIP60 isoform 2 (TIP60α), and TIP60 isoform 3 (TIP60β). iTIP60 retains the alternatively spliced intron 1 [30]. TIP60β lacks exon 5 [31]. Different isoforms potentially involve distinct functions in the cells. When tested in the yeast two-hybrid, all three TIP60 isoforms interacted with GAL4BD-SseF chimerical protein (Fig. 1). To determine the region of SseF that is responsible for interacting with TIP60, a series of SseF deletions was constructed and tested in the yeast two-hybrid for their ability to interact with TIP60. We found that amino acids 50-66 were sufficient for mediating the SseF and TIP60 interaction (Fig. 1). We observed weak interactions occasionally when confirming the interactions biochemically using purified recombinant proteins. This is not unusual as most wild-type enzymes do not interact strongly with their target molecules. It is also possible that the three putative transmembrane regions in SseF are essential for tight interactions and the fragment devoid of the transmembrane regions has reduced affinity rendering it difficult to detecting the interactions *in vitro*.

### SseF increases the histone acetylation activity of TIP60

TIP60 is a multifunctional acetyltransferase involved in many transcriptional regulations by serving as a co-regulator [5]. The interaction of SseF with TIP60 suggested that SseF may serve as the substrate for TIP60-mediated acetylation. To test whether SseF serves as the substrate for TIP60, an *in vitro *HAT assay was conducted, using purified recombinant MBP-TIP60 as acetyltransferase and GST-SseF^1-66 ^as the substrate [2,4,5]. When probed with antibodies specific for acetylated species, adducts were detected when histone was added to the reaction in the presence of MBP-TIP60 (**data not shown**). No SseF acetylation was observed when GST-SseF^1-66 ^was used in the reaction. Similar results were obtained when partially enriched full-length SseF was used in the reaction (**data not shown**). Thus, SseF is not likely the substrate for TIP60.

**Figure 2:**
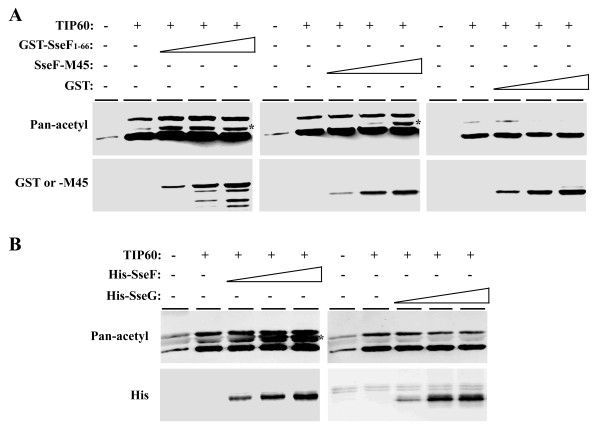
**SseF increases the histone acetylation activity of TIP60**. HAT assays were performed using recombinant MBP-TIP60 protein as acetyltransferase and histone as the substrate in the presence of (**A**) GST-SseF^1-66^, SseF-M45, GST, or (**B**) His-SseF, His-SseG. Acetylated histones were detected by Western blot using the pan-acetyl antibody. Total amounts of proteins were examined by Western blot using anti-GST, -M45, or His antibodies, respectively. * Indicate acetylated histone isoform H2.

Since SseF is not likely the substrate for TIP60, we explored the possibility that SseF-TIP60 interaction may alter the acetylation activity of TIP60 without direct modification. We then examined whether GST-SseF^1-66 ^affected TIP60-mediated histone acetylation, using the *in vitro *HAT assay with recombinant MBP-TIP60 as the acetyltransferase and histone as the substrate in the presence of GST-SseF^1-66 ^or GST. We observed an increase in the amount of acetylated histone H2, H3 and H4 when GST-SseF^1-66 ^was added to the reaction while addition of GST had no obvious effect (Fig. 2A). The increase is more pronounced for the histone isoform H2 and more moderate for isoforms H3 and H4 (Fig. 2A) [2]. We next explored whether the full-length SseF has similar effect as the GST-SseF^1-66 ^to histone acetylation. We previously showed that SscB is the chaperone for SseF and that they interact with each other [20]. We obtained SseF-M45 by co-expressing SseF and SscB followed by pulling down His-SscB. The enriched SseF-M45 was then used in the *in vitro *HAT assay as described above. Again, we observed increased TIP60-mediated Histone H2 acetylation in the presence of SseF-M45 (Fig. 2A). Similar enhancement of TIP60-mediated histone H2 acetylation was noted when enriched His-SseF was used in the HAT assay (Fig. 2B). No obvious change in TIP60-mediated histone acetylation was found when His-SseG was used in the same reaction (Fig. 2B). Taken together, we conclude that SseF can potentiate the Histone H2 acetylation activity of TIP60.

### TIP60 protein level is increased upon *Salmonella *infection

**Figure 3:**
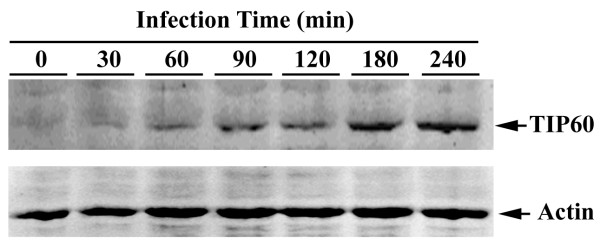
**TIP60 is up regulated upon *Salmonella *infection**. HeLa cells were infected with wild-type *Salmonella *for the indicated time intervals. Infected cell lysates were subjected to SDS-PAGE followed by Western blot using anti-TIP60 antibody (upper panel). Actin levels in the same samples were also determined as a control (lower panel).

TIP60 is known to be involved in diverse functions and the endogenous basal level of TIP60 is usually low. TIP60 level increases significantly upon UV irradiation [32]. Upon *Salmonella *infection of HeLa cells, we observed an increase in TIP60 as short as 60 minutes after infection and approaching maximum induction three hours post infection (Fig. 3). Actin levels in the same samples remained constant up to 4 hours after infection. This supports the notion that TIP60 might play an important role during *Salmonella *infection. This increase is SseF-independent, as similar increase was also observed when infected with an *sseF *mutant *Salmonella *strain and TIP60 was not concentrated at the vacuoles (data not shown). SseF was not detected in infected cells possibly due to the low amounts translocated during *Salmonella *infections.

### TIP60 is required for efficient intracellular *Salmonella *replication

**Figure 4:**
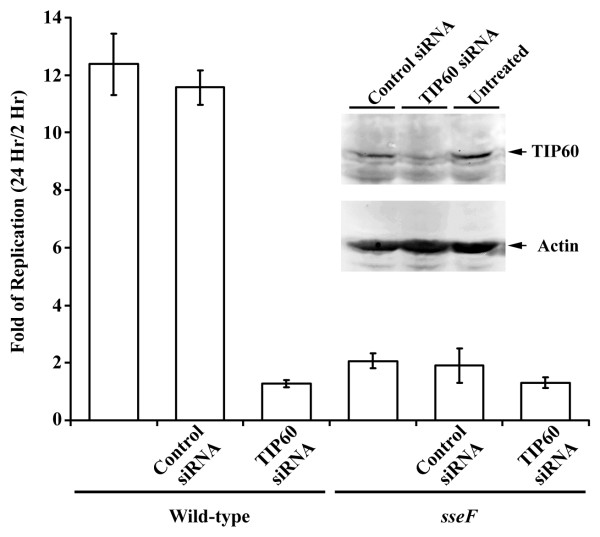
**TIP60 is required for efficient *Salmonella *replication**. Transfected macrophages were infected with wild-type *S. typhimurium *or the *sseF *mutant strains at an MOI of 10. Extracellular bacteria were removed by washing and gentamicin treatment. At 2 and 24 h after bacterial invasion, cells were lysed, and the number of intracellular bacteria was enumerated. The data shown were obtained from three independent experiments with standard errors. The effect of TIP60 knockdown is verified by Western blot using the anti-TIP60 antibodies. Actin was used a control.

Previous studies have shown that SseF is required for efficient intracellular *Salmonella *replication in macrophages [10]. Since TIP60 acetyltransferase interacts with SseF, TIP60 might be required for efficient intracellular *Salmonella *replication. To test this, we used siRNA to down-regulate the endogenous level of TIP60. Macrophages were transfected with a plasmid expressing TIP60 siRNA or a control vector expressing the scrambled siRNA. As shown in Fig. 4, TIP60 siRNA effectively suppressed the endogenous TIP60 expression, while the control siRNA did not. Transfected macrophages were infected with wild-type *S. typhimurium *or the *sseF *mutant strains. As shown in Fig. 4, down-regulation of TIP60 leads to less efficient *Salmonella *replication comparable to the level of *sseF *mutant strain [10]. There was not significant replication change in cells expressing the scrambled siRNA. These data support our notion that TIP60 is required for efficient intracellular *Salmonella *replication in macrophages.

## Discussion

We do not know yet the molecular mechanism of how SseF and TIP60 interaction affects the SCV and intracellular *Salmonella *replication. Ideally, a mutant SseF lacking the TIP60-binding domain can be used to assess the requirement for SseF-TIP60 interaction for its function, however such a mutant is defective in secretion and thus not translocated, making it impossible to assess its effect during infection. Definitive identification of the acetylation site and subsequent characterization of proper mutants lacking TIP60-mediated acetylation will be required to validate this hypothesis. Alternatively, SseF-TIP60 interaction may alter the acetylation activity of TIP60, thus affecting TIP60 related functions. Supporting this hypothesis, our preliminary *in vitro *acetylation assays suggest that SseF increased the histone acetylation activity of TIP60, especially for histone H2. Histone is the only known substrate for Tip60. Total histone acetylation was not increased in infected cells (data not shown). This is consistent with the low amount of SseF translocated. It is possible that local SseF concentration may be higher in infected cells.

Although TIP60 is not known to be directly involved in vesicle trafficking, it is possible that TIP60 affected histone acetylation leading to altered expression of trafficking-related proteins. Interestingly, our preliminary data showed that knock down of TIP60 reduced continuous Sif formation, a phenotype similar to that of the *sseF *null mutant (Additional file 1: Fig. S1). Future experiments are required to determine whether the increase in histone acetylation leads to increases in TIP60-mediated downstream functions. This may ultimately help us to understand how SseF interact with TIP60 to promote *Salmonella *replication inside the host cells.

## Conclusions

We found that TIP60, an acetyltransferase, interacts with *Salmonella *SseF. We further showed that the TIP60 acetylation activity was increased in the presence of SseF, and TIP60 was upregulated upon *Salmonella *infection. More importantly, TIP60 is required for efficient intracellular *Salmonella *replication in macrophages. Our study demonstrated that *Salmonella *may use SseF to exploit the host TIP60 acetyltransferase activity to promote efficient *Salmonella *replication inside host cells.

## Competing interests

The authors declare that they have no competing interests.

## Authors' contributions

XW generated figure 1, 2, 3, 4. DL contributed to figure 4. DQ and DZ directed the project and analyzed data. All authors read and approved the final manuscript.

## Supplementary Material

Additional file 1 **TIP60 is required for continuous *Salmonella*-induced filament formation**. HeLa cells were transfected with a plasmid expressing TIP60 siRNA or a control vector expressing the scrambled siRNA. Transfected cells were infected with wild-type Salmonella. Infected cells were stained for TIP60 (red) or LAMP2 (green). Arrows indicates Sifs, and arrowheads indicate the "pseudo-Sifs". Click here for file
