# Supplementary figures and images for: Involvement of TIP60 acetyltransferase in intracellular Salmonella replication
Source: BMC Microbiol. 2010 Aug 26;10:228. doi: 10.1186/1471-2180-10-228 (PMC3313078; doi:10.1186/1471-2180-10-228)

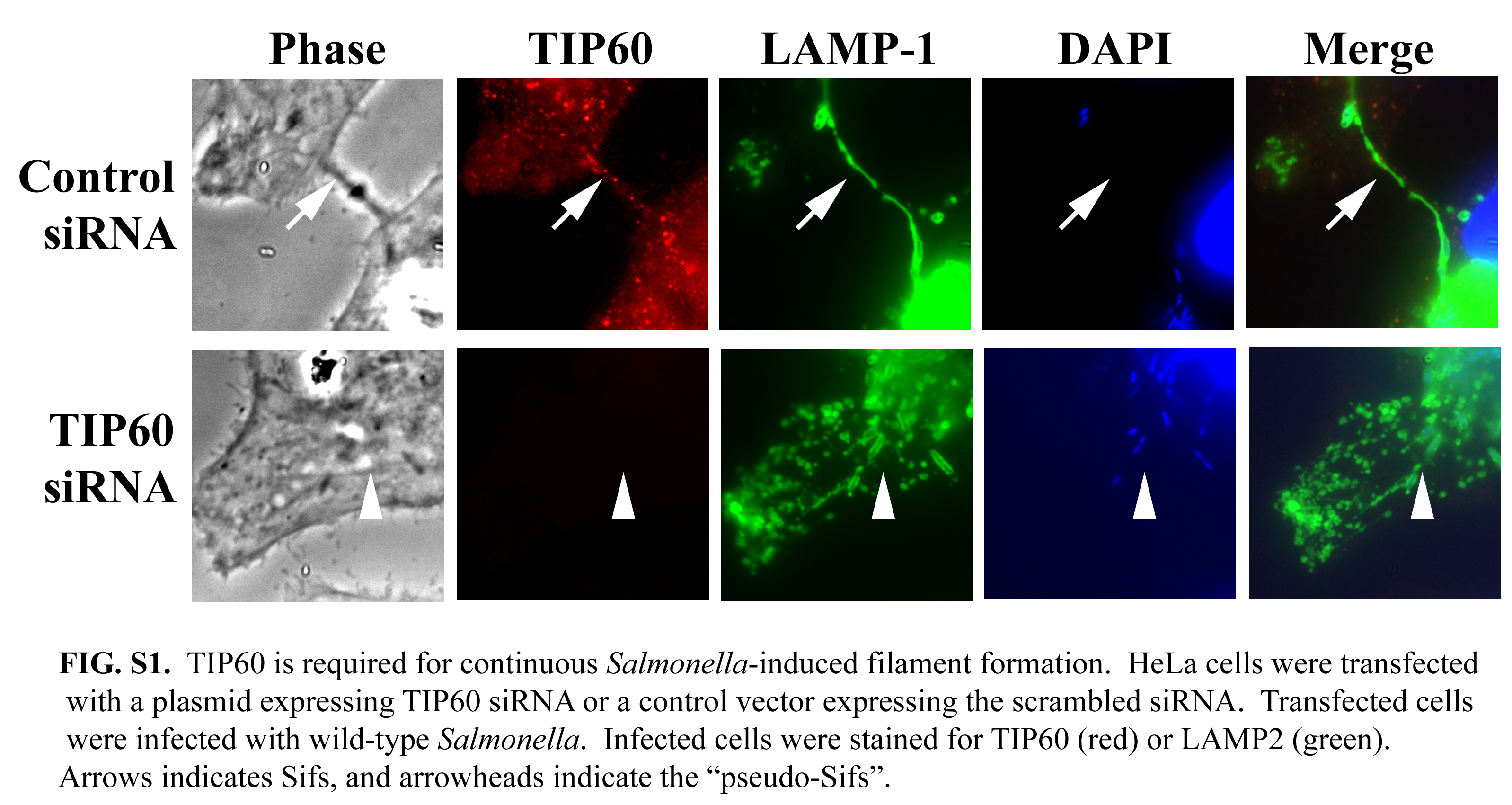

Supplement: Additional file 1 — TIP60 is required for continuous Salmonella-induced filament formation. HeLa cells were transfected with a plasmid expressing TIP60 siRNA or a control vector expressing the scrambled siRNA. Transfected cells were infected with wild-type Salmonella. Infected cells were stained for TIP60 (red) or LAMP2 (green). Arrows indicates Sifs, and arrowheads indicate the "pseudo-Sifs". [file 1471-2180-10-228-S1.TIFF]
